# The Cytotoxicity and Genotoxicity of Three Dental Universal Adhesives—An In Vitro Study

**DOI:** 10.3390/ijms21113950

**Published:** 2020-05-31

**Authors:** Adam Wawrzynkiewicz, Wioletta Rozpedek-Kaminska, Grzegorz Galita, Monika Lukomska-Szymanska, Barbara Lapinska, Jerzy Sokolowski, Ireneusz Majsterek

**Affiliations:** 1 Department of Clinical Chemistry and Biochemistry, Medical University of Lodz, 90-419 Lodz, Poland; adam.wawrzynkiewicz@stud.umed.lodz.pl (A.W.); wioletta.rozpedek@umed.lodz.pl (W.R.-K.); grzegorz.galita@umed.lodz.pl (G.G.); 2 Department of General Dentistry, Medical University of Lodz, 90-419 Lodz, Poland; monika.lukomska-szymanska@umed.lodz.pl (M.L.-S.); barbara.lapinska@umed.lodz.pl (B.L.); jerzy.sokolowski@umed.lodz.pl (J.S.)

**Keywords:** dental materials, dental universal adhesives, cytotoxicity, genotoxicity, flow cytometry

## Abstract

Dental universal adhesives are considered an useful tool in modern dentistry as they can be used in different etching techniques, allow for simplified protocol and provide sufficient bond strength. However, there is still no consensus as to their toxicity towards pulp. Thus, the present study aimed to evaluate the cytotoxicity and genotoxicity of three universal adhesives: OptiBond Universal, Prime&Bond Universal and Adhese in an in vitro experimental model, monocyte/macrophage cell line SC (ATCC CRL-9855). The cytotoxicity was measured by means of XTT assay, whereas the genotoxicity (comet assay) was evaluated based on the percentage of DNA present in the comet tail. Furthermore, the ability of the adhesives to induce apoptosis was analyzed using flow cytometry (FC) with the FITC annexin V/propidium iodide (PI) double staining. The analysis of the cell cycle progression was performed with FC using PI staining. OptiBond Universal presented significant, while Prime&Bond Universal and Adhese Universal had minimal cytotoxicity and genotoxicity towards human SC cells. Moreover, only OptiBond Universal increased the level of apoptosis in SC cell line. None of the adhesives showed significant cell cycle arrest, as revealed by FC analysis. Due to substantial differences in toxicity in in vitro studies of dental adhesives, there is a great need for further research in order to establish more reliable test protocols allowing for standardized methodology.

## 1. Introduction

Adhesive dentistry constitutes one of the major branches of the dentistry that is mainly focused on the development of materials that establish an effective bond with tooth tissue. Inasmuch, dental adhesive systems have grown in popularity worldwide and nowadays attract a significant research interest [1,2]. Dental adhesion is commonly used in almost all dental specialties, since adhesive dentistry constitutes a crucial key to minimally invasive, esthetic and tooth-preserving dental restorations [3]. The effectiveness of adhesive bonding is directly correlated with the chemical composition of the adhesive, appropriate clinical handling of the material and knowledge of the morphologic changes, that can be caused by various bonding procedures on dental tissue [1]. The major components of the adhesive systems constitute acrylic resin monomers, organic solvents, initiators, inhibitors and sometimes filler particles. Each one of the above-mentioned components has a specific function. Detailed characterization of the chemical properties and the adhesives components as well as their biologic impact on dental tissue are a key to understand or predict their behavior within the organisms. Interestingly, based on both proportional composition as well as the chemistry of these components dental adhesives may be divided into different generations [1,4]. Dental adhesives, that constitute a multifunctional systems, may be used as self-etch adhesives, etch-and-rinse adhesives or in a selective enamel etching technique [5,6,7,8,9]. They are commonly used for a wide range of clinical application, whereas a several important factors should be considered before selection of the bonding procedure and adhesive system for the vital dentine. In this case longevity of the restoration, lack of secondary caries and of pulp damage both due to eluted monomers and bacteria and their products, that may penetrate developing gaps between material and cavity walls, should be combined [1]. Manufacturers still change the composition and properties of dental adhesives to increase the longevity of restoration. The major factors affecting the bonding durability of dental adhesives include not only the type of solvent, the chemical components, their molecular weights or the pH associated with proper preparation of the tooth surface, but also additional components, added to improve bonding durability, including collagen crosslinking agents, antioxidants and protease inhibitors [10].

The ever-increasing demand for simplified adhesive systems has resulted in the development of a next generation dental adhesives termed universal adhesives. However, the development of universal adhesive systems constituted a great innovation in adhesive dentistry, it is still unknown whether universal adhesive systems may be use in all adhesive procedures, since their detailed characterization is still required [11]. Generally, dental universal adhesives may be defined as single-bottle, no-mix systems [12]. Universal adhesives are characterized by simplified application steps and capability of bonding to various materials and dental hard tissues after appropriate surface treatment [13,14]. In general, they are composed of complex mixtures of crosslinked and functional hydrophilic and hydrophobic monomers in appropriate solvents, usually comprising acetone, ethanol, and/or water [4]. The monomers are capable of producing chemical and micromechanical bond to the dental substrates [5,6]. Adhesives comprise of specific carboxylate and/or phosphate ionic monomers, that facilitate bonding to calcium found in hydroxyapatite [15,16,17]. Apart from that, universal adhesives also contain the other compounds such as biphenyl dimethacrylate (BPDM), dipentaerythritol pentaacrylate phosphoric acid ester (PENTA) and polyalkenoic acid copolymer that may facilitate the adhesion to the tooth structures. Universal adhesives also use the combination of monomers such as hydrophobic decanediol dimethacrylate (D3MA), hydrophilic 2-hydroxyethyl methacrylate (HEMA) and intermediate bisphenol A-glycidyl methacrylate (bis-GMA) [2]. Hydrophobic ends of these monomers both combine with the restorative materials and link with each other, while hydrophilic ends adhere to the hard dental tissue. The functional monomers present in the adhesives’ formulation allow for various etching techniques and they also enhance the bond strength. These functional monomers, such as methacryloyloxi-decyl-dihydrogen-phosphate (MDP), N-Phenyl-p-phenylenediamine (Phenyl-P) or 4-methacryloxyethyl trimellitic acid (4-MET), undergo chemical interaction with tooth hydroxyapatite which results in formation of nanolayers [8]. The nanolayers that are formed most effectively with MDP, are strongly hydrophobic. Thereby, their hybrid layer is less susceptible to hydrolysis that increases the bond strength to dentin [8,18]. Nanolayers of stable MDP–calcium salts are formed and deposited at various degrees and quality, depending on the type of adhesive system [8,19]. The terminal ends of MDP present hydrophilic properties at first, but become more hydrophobic after polymerization and interaction with dentin [9]. MDP has a capability to bond various surfaces such as dentin [20], titanium, metal alloys [21,22] or polycrystalline ceramics [23,24,25]. The incorporation of silane into the universal adhesive’s composition simplifies the cementation procedure. It eliminates the silanization step during the placement of resin composites, glass ceramics, zirconia or metal restorations directly or indirectly [15,26].

Due to the fact that dental adhesives are in direct contact with hard and soft dental tissues, their biocompatibility is extremely important [27]. There are claims of using dental adhesives in direct contact with the pulp, as the means of direct pulp cupping. In this treatment method, the dental adhesives showed no hard tissue formation induction in comparison to Ca(OH)_2_ cements [28,29,30,31]. There was also no expression of type 3 collagen and fibronectin, which were present in teeth treated with Ca(OH)_2_ cements [29]. According to Paula et al., dental adhesive systems may cause a potential pulp tissue damage and they do not recommend using this materials in the direct pulp capping techniques [32]. Scientific data concerning the biocompatibility of universal dental adhesives is contradictory. Most authors believe that universal adhesives cannot be used for direct pulp capping due to the reported inflammation [33,34,35]. On the other hand, universal adhesives, despite simplified clinical procedures and improved adhesion presented cytotoxic effect towards human cells such as gingival fibroblasts [36]. The main irritants found in dental adhesives constitute monomers, certain amount of which is left unpolymerized even after light curing [37]. Components of adhesive systems, such as Bis-GMA, urethane dimethacrylate (UDMA), triethylene glycol dimethacrylate (TEGDMA), camphorquinone, HEMA and some other compounds, showed cytotoxicity when placed in contact with mammalian fibroblasts [37]. Mentioned monomers diffuse through the dentinal tubules in concentrations that induce a toxic effect on pulp [38].

In order to precisely define the biologic properties of the investigated material in in vitro experimental models, various research methods should be used including cytotoxicity and genotoxicity assays, apoptosis detection tests as well as evaluation of the cell cycle progression. Both in vitro and in vivo studies give an opportunity for the evaluation of different characteristics of the material [39,40,41]. Studies that are performed to evaluate the cell damage [42,43,44] and genotoxicity of the dental materials are commonly performed in vitro using human leukocytes as a reference cell type [45,46,47]. Cytotoxicity of the dental adhesives was widely described in the literature with the use of assays based on reduction of tetrazolium salts such as Cell Proliferation Kit II (XTT) and I (MTT), which is recommended by international standards such as ISO 10,993 [48]. Nowadays, the new paradigms of product safety assessment were established based on the mechanisms involved in cytotoxic pathways rather than on cell death exclusively [49]. In biomaterial research there are several methods used that include the investigation of apoptosis, necrosis [50,51] and cell cycle analysis via the flow cytometry (FC) [51,52,53]. FC is a relatively new method used in dental material research and to date, only few dental materials were investigated using the above-mentioned laboratory technique. Previous studies showed that FC may also be used in the evaluation of antibacterial properties of the dental adhesives [54,55]. The other method that was used in recent studies is a comet assay, which is suitable for detecting DNA damage at the level of individual eukaryotic cells. Due to its high sensitivity, it was used to evaluate genotoxicity of various agents [56,57].

With regards to all mentioned aspects, the main aim of the present research constitutes a detailed characterization of the properties of dental universal adhesives in a highly standardized, in vitro experimental model.

## 2. Results

### 2.1. Analysis of the Cytotoxicity of the Dental Universal Adhesives

**Figure 1:**
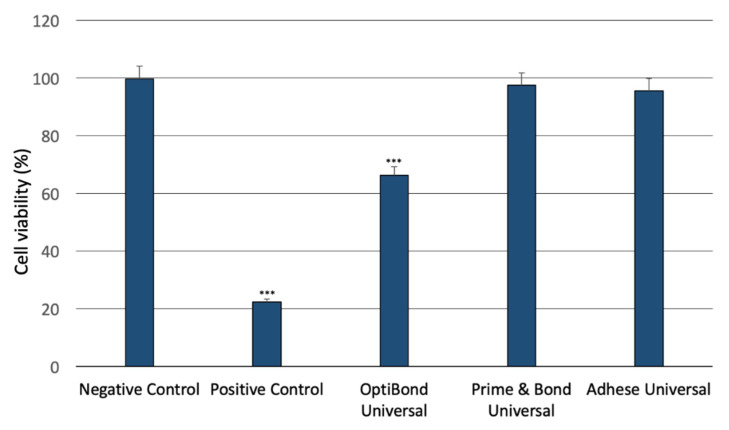
Cytotoxicity of the investigated adhesives. *** *p* < 0.001 versus negative control.

Obtained XTT assay outcomes showed significant differences in the cytotoxic properties of the investigated compound eluates. Monocyte/macrophage peripheral blood SC (ATCC CRL-9855) cells were incubated with the investigated compounds for 24 h. The obtained results showed that only OptiBond Universal significantly decreased their viability. Both in the case of Prime&Bond Universal and Adhese Universal, no significant changes of cell viability were observed (Figure 1).

### 2.2. Analysis of the Genotoxicity of the Dental Universal Adhesives

**Figure 2:**
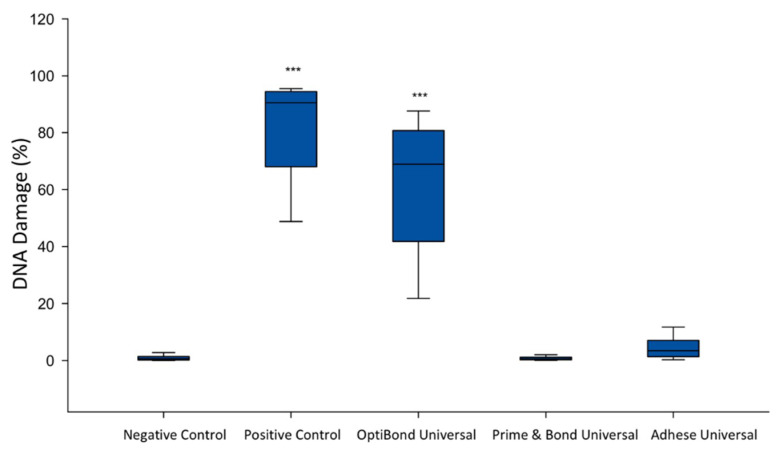
Genotoxicity of the investigated adhesives. *** *p* < 0.001 versus negative control.

The level of DNA damage was estimated using the alkaline version of the comet assay. The alkaline comet assay enables detection of oxidative DNA damage, single- and double-stranded breaks—as well as presence of alkaline labile sites. The amount of DNA damage was assessed based on the percentage of DNA in the comet tail. The significant increase in DNA damage in SC cell line for OptiBond Universal was observed after 24 h incubation. Both Prime&Bond Universal and Adhese Universal did not induce a significant DNA damage in the tested cell line (Figure 2).

### 2.3. Apoptosis Detection by FITC Annexin V/PI Double Staining of the Dental Universal Adhesives

**Figure 3:**
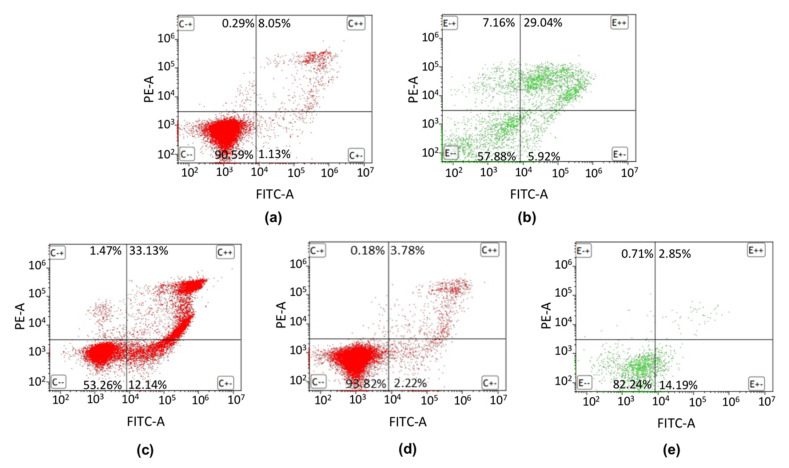
Flow cytometric FITC annexin V/propidium iodide (PI) double staining analysis of apoptosis. (**a**) negative control; (**b**) positive control; (**c**) OptiBond Universal; (**d**) Prime&Bond Universal; (**e**) Adhese Universal. Dot plot graphs indicate the percentage of viable (FITC annexin V negative, PI negative), early apoptotic (FITC annexin V positive, PI negative) late apoptotic (FITC annexin V positive, PI positive) and necrotic (FITC annexin V negative, PI positive) cells.

In order to assess activation of apoptosis in the SC cell line after exposure to OptiBond Universal, Prime&Bond Universal and Adhese Universal, the FITC annexin V/propidium iodide (PI) double staining and subsequently FC analysis were performed. The 34.96% of SC cells treated with 1-µM staurosporine for 16 h underwent apoptosis than control cells cultured in complete medium for 24 h. After 24 h incubation, only in the case of OptiBond Universal the apoptosis was induced in SC cells (approximately 45% of cells were at the early and late stages of apoptosis) (Figure 3). Prime&Bond Universal and Adhese Universal did not evoked a significant activation of apoptotic cell death in the investigated cell line. In addition, there was no increase in the level of necrotic cells after exposure to each of the tested adhesive systems.

### 2.4. Analysis of the Cell Cycle Progression by PI Staining of the Dental Universal Adhesives

**Figure 4:**
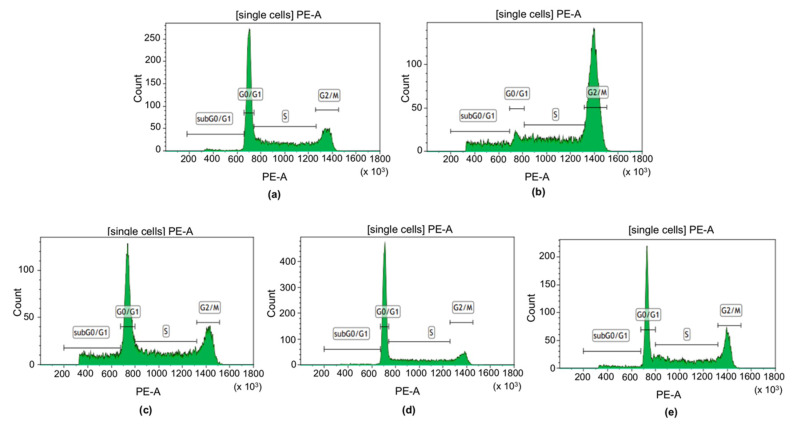

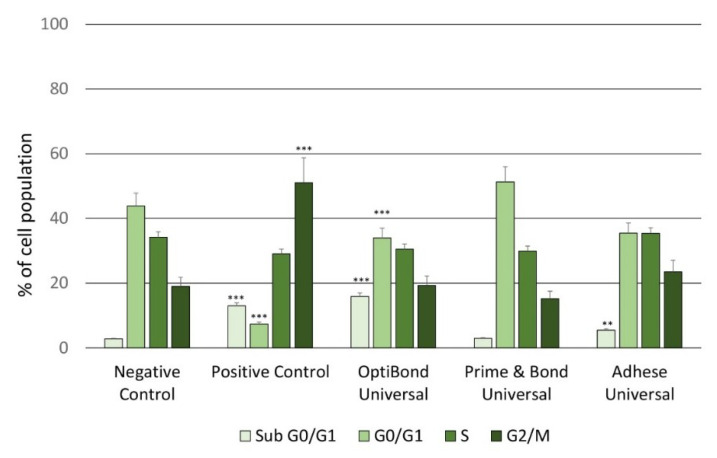
Flow cytometry (FC) analysis of cell cycle progression. (**a**) negative control; (**b**) positive control; (**c**) OptiBond Universal; (**d**) Prime&Bond Universal; (**e**) Adhese Universal. ** *p* < 0.01, *** *p* < 0.001 versus negative control.

The cell cycle distribution of PI-stained SC cell line after 24 h exposure to OptiBond Universal, Prime&Bond Universal and Adhese Universal was analyzed by FC. As predicted, cell cycle of SC cells exposed to 1 μM nocodazole was arrested at G2/M phase. The cell cycle progression of SC cells treated with the investigated compounds was similar to SC cells cultured in the complete medium. Thereby, none of the tested compounds induced a significant G2/M cell cycle arrest in SC cells (Figure 4).

## 3. Discussion

The dental adhesives have to be examined with highly standardized experimental model with regards to their direct contact with dental tissues. Scientific data concerning the biocompatibility of dental universal adhesives is contradictory. For instance, several research data indicate that the universal adhesives could not be used for direct pulp capping due to the reported inflammation [33,34,58]. Nevertheless, to date there are no studies that evaluated the biocompatibility of dental universal bonding systems using in vitro tests, as presented in this research. The application of the novel techniques such as XTT assay, comet assay, analysis of the level of apoptosis and cell cycle distribution via the FC may lead to the introduction of new testing standards in dental materials science.

The results of the present study regarding the biocompatibility of universal bonding systems showed consistency in all conducted tests. Prime&Bond Universal and Adhese Universal presented minimal cytotoxicity and genotoxicity towards human SC cells in colorimetric XTT assay. Interestingly, OptiBond Universal presented significant cytotoxicity and genotoxicity towards SC cell line. Moreover, only OptiBond Universal showed significant ability to induce apoptosis in SC cell line. On the other hand, none of the tested adhesives showed significant cell cycle arrest in G2/M phase in FC analysis. We suggest that the differences in toxicity of the tested dental bonding systems may resulted from their composition, i.e., acidic monomers and other compounds.

The biocompatibility assessment is crucial for the clinical validation of dental materials and to date, only several studies investigated the cytotoxicity and genotoxicity of dental bonding systems [59,60]. In vitro studies of biocompatibility are important as well as allow the evaluation of many samples simultaneously. Moreover, only materials that appear to be efficacious may undergo analysis in in vivo experimental models. The cell culture assays provide controllable and repeatable method of assessment and are ethically more acceptable in comparison to in vivo animal studies, more important, the results may lead to significant clinical conclusions in biomaterial research [61,62].

There are different cell types that may be used in in vitro studies on cytotoxicity and genotoxicity. Most studies regarding the toxicity of dental bonding systems were conducted on cells derived from pulp or soft tissues of the oral cavity [63], whereas cytotoxicity assessment of the dental bonding systems were mostly performed with the primary immortalized or commercially available cell lines [50,51,52,59,63,64,65,66,67]. Evaluation of cytotoxicity and genotoxicity of resin-based dental materials conducted on several composites, including Tetric EvoCeram, Tetric EvoFlow, Filtek Ultimate, Filtek Ultimate Flow, G-aenial and G-aenial Flow, demonstrated that cured forms of this materials presented no significant cytotoxicity and genotoxicity, while uncured materials proved toxic towards human lymphocytes. The authors concluded that it may occur due to the higher amount of free monomers in uncured composites [68]. Other study investigated the cytotoxicity using the dentin barrier test device and three-dimensional cell culture. They demonstrated that total-etch adhesive presented significant cytotoxicity which varied depending on the dentin thickness. However, the self-etch adhesives used in the study were non-cytotoxic in all dentin thickness models tested [65]. Other studies were conducted on mouse odontoblast cell line (MDPC-23) and included 6 Adper Easy Bond, Xeno V, iBond, AdheSE One, Clearfil SE primer and Adper Single Bond 2 adhesive systems. All of these adhesives showed marked increase in apoptotic activity as well as examination under scanning electron microscope presented cytoplasmic membrane shrinkage and residual membrane fragments from dead cells [51]. Some other studies, in the field of dental bonding systems usage in endodontics, presented a significant cytotoxic effect of Clearfil Universal and Adper Scotchbond-multipurpose adhesive systems towards human gingival fibroblast cell line [64].

However, cells cultured in vitro for many generations undergo genomic transformations and/or mutations and thus are not reliable for studies regarding the DNA damage. As far as genotoxicity is concerned, the most preferable studies are those using the diploid cell lines such as human leukocytes [69]. In common sense, we chose the SC cell line as the preferable in both cytotoxicity and genotoxicity studies that were performed in this research.

In recent years there were limited studies on dental bonding systems by means of highly sensitive comet assay, which showed insignificant increase of their genotoxicity in human blood cells [70]. Therefore, present results cannot be compared with the other studies. However, several studies showed that the leachability of components of the dental bonding systems, after their incomplete conversion, may cause the rise in their cytotoxicity [52,53,63,64]. Scientific data suggested that the dental bonding systems may contain different combinations and different concentrations of methacrylate monomers such as Bis-GMA, TEGDMA, HEMA, UDMA and PENTA. Therefore, it is possible that the variation in concentrations affect the toxicity of each material. Interestingly, the synergistic interaction between the components of the dental bonding systems may result in the greater cytotoxic effect than the individual components [71].

Previous studies showed that bis-GMA presents the highest cytotoxicity followed by TEGDMA, UDMA and HEMA, which are, on the other hand, moderately cytotoxic [71,72,73,74,75,76]. The presence of methacrylate monomers such as PENTA and UDMA is possibly the major cytotoxic factor found in dental adhesive systems [77]. Bis-GMA is characterized by relatively high toxicity, whereas due to its higher molecular weight it presents low ability to penetrate the dentin [78]. Moreover, bis-GMA undergoes hydrolysis, which releases of water soluble metabolites such as methacrylic acid. This could result in a loss of cell membrane permeability via induction of TNF-α release or alteration in lipid layer [79].

It was demonstrated that typical components of dental bonding systems as well as restorative materials, such as HEMA and TEGDMA, are able to spread through the dentin tubules and thus they reach the pulp tissue at millimolar concentrations. Even negligible levels of mentioned monomers demonstrated an ability to arrest the proliferation of pulp cells [80,81]. Other studies also revealed that HEMA and TEGDMA are detrimental to odontogenic differentiation of pulp stem cells. This in turn negatively affects pulp tissue homeostasis and repair capabilities [82,83,84,85].

Some studies presented that the cytotoxic effect of dental bonding systems and resin monomers are linked to the cell cycle arrest at specific cell cycle phases [77,86,87]. TEGDMA caused the reduction of the proliferation rate in human gingival fibroblasts, by inducing arrest at G2/M phase of the cell cycle [87]. Our study showed no significant arrest of the cell cycle progression despite the fact that the evaluated universal adhesives contained monomers that demonstrated the ability to arrest the cell cycle progression in culture model *in vitro*. In other studies, dental adhesives containing MDP showed varied cytotoxic effect, ranging from significant cytotoxicity for Clearfil Liner Bond 2 V, than ED Primer II [59], to the mild cytotoxicity effect, as presented by Clearfil Protect bond [73].

The presented study has a specific limitation. The in vitro model shows limited information on the number of residual monomers and other components in the mixture of the adhesives that can penetrate through the dentinal tubules to the dental pulp. There are several variables that affect this leaching including the thickness of dentine, exposed surface area, presence of smear layer [59,88]. The in vitro studies could not replicate the clinical performance of the materials as they can be present in the organism for several years [89]. Thus, the in vitro model does not take into consideration the long-term effects, such as distribution through the dental tubules towards the pulp and the immune response present in human tissues [90]. The comparison of the in vitro effect and clinical performance of the dental materials must be investigated in further studies. However, based on the obtained data in this study, we strongly suggest that in vitro cytotoxicity and genotoxicity evaluation should be introduced into biomaterials research as a key safety marker for the assessment of dental materials.

## 4. Materials and Methods

In the present study, three universal bonding systems were analyzed: OptiBond Universal, Prime&Bond Universal, Adhese Universal (Table 1).

### 4.1. Cell Line and Eluate Preparation

All investigations were performed in in vitro experimental model using a commercially available monocyte/macrophage peripheral blood cell line—SC (ATCC CRL-9855) purchased from the American Type Culture Collection (ATCC; Manassas, VA, USA). Cell culture were maintained under standard conditions (37 °C; 5% pCO_2_; 95% humidity), according to the guidelines provided by the vendors. Cells were cultured in Iscove’s Modified Dulbecco’s Medium (IMDM) with 4-mM l-glutamine adjusted to contain 1.5 g/L sodium bicarbonate and supplemented with 0.05-mM 2-mercaptoethanol, 0.1-mM hypoxanthine and 0.016-mM thymidine (90%); fetal bovine serum (10%).

The amount of 50 µL of each investigated dental bonding system was placed in round bottom of Eppendorf tubes and polymerized (LED lamp intensity over 1000 mw/cm^2^, The CURE-TC-01, Spring Health Products, PA, USA) according to the manufacturer’s instructions. Then 1 mL of medium was added and incubated for 24 h at 37 °C. The obtained eluate, after centrifugation, was prepared for further experiments.

### 4.2. Cytotoxicity Analysis

The cytotoxicity of the investigated compounds was measured using the XTT colorimetric assay (Thermo Scientific, Waltham, MA, USA). XTT is used to assess cells’ viability as a function of redox potential. Actively metabolizing cells reduce the tetrazolium salt (XTT) to an orange water-soluble formazan. All of the experiments were performed in triplicate with similar results. Test samples were prepared in 96-well plates by adding up to 50 µL (8 × 10^3^ cells/well) of cell suspension in complete medium, 50 µL of prepared eluate. The positive control constitutes cells suspended in 96% isopropyl alcohol, that high concentrations are highly toxic to cells leading to their lysis, whereas the negative control cells cultured in a complete medium. The analyzed samples were incubated for 24 h. Subsequently, 25 µL of XTT/PMS mixture was added to each well. After 4 h incubation, absorbance was measured at a wavelength of 450 nm using Synergy HT (BioTek) spectrophotometer.

### 4.3. Genotoxicity Assessment

The genotoxicity of analyzed compounds was assessed using a comet assay. Comet assay is a sensitive and rapid technique for quantifying and analyzing DNA damage in individual cells. Assays were prepared in 12-well plates by adding 5 × 10^4^ cells in 500 µL of complete medium and 500 µL of previously prepared eluates. Cells suspended in 100% DMSO (Sigma-Aldrich Corp., St. Louis, MO, USA), that high concentrations are highly toxic to cells leading to their lysis, were used as a positive control, whereas cells suspended in 1 mL of complete culture medium as a negative control. All samples were incubated for 24 h. Cell suspension in 0.37% LMP agarose (Sigma-Aldrich Corp., St. Louis, MO, USA) was placed on microscope slides previously coated with NMP agarose (Sigma-Aldrich Corp., St. Louis, MO, USA). Preparations were incubated in lysis buffer at pH 10 (2.5-M NaCl, 10-mM Tris, 100-mM EDTA) containing TritonX-100 (Sigma-Aldrich Corp., St. Louis, MO, USA) at a final concentration of 1% at 4 °C for 60 min. After 1 h incubation, the preparations were incubated 20 min in development buffer (300-mM NaOH, 1-mM EDTA) at 4 °C, followed by electrophoresis (32 mA, 17 V, 20 min) at 4 °C in electrophoretic buffer (30-mM NaOH, 1-mM EDTA). After staining with a DAPI fluorescent dye, preparations were analyzed under a fluorescent microscope. Cell damage was evaluated based on the percentage of DNA in the comet tail.

### 4.4. Apoptosis Detection

Apoptotic cell death induced by filtrates of test compounds was assessed using FITC Annexin V Apoptosis Detection Kit I purchased from BD Pharmingen™ (ApoAlert Annexin V, Clontech, California, USA). This method is based on high affinity of Annexin V to phosphatidylserine which, as a result of induction of apoptosis, is translocated to the outer parts of the cell membrane as well as on the propidium iodide (PI) that constitutes a marker of cell membrane permeability. SC cells (1 × 10^6^ cells/well) plated on 12-well plates were incubated with previously prepared compounds filtrates diluted in ratio 1:1 with medium for 24 h. Cells treated with staurosporine (Sigma-Aldrich Corp., St. Louis, MO, USA) at a concentration of 1 µM for 16 h constituted a positive control, whereas a negative control cells suspended in complete culture medium and incubated for 24 h. Subsequently cells were washed with cold PBS (Sigma-Aldrich Corp., St. Louis, MO, USA) twice and double stained with annexin V, as a marker of early apoptosis and PI as a marker of cell membrane disintegration, necrosis and late apoptosis. The percentage level of apoptotic cells was analyzed by FC using the Beckman Coulter CytoFLEX. The obtained data were analyzed using the Kaluza analysis 1.5 A software (Beckman Coulter).

### 4.5. Cell Cycle Analysis

Cell cycle analysis was carried out by FC using the propidium iodide (PI) staining. Cells were seeded on 12-well plates (1 × 10^6^ cells/well) and incubated with the previously prepared compounds eluate diluted in ratio 1:1 with medium for 24 h. Cells treated with 1 µM nocodazole (Sigma-Aldrich Corp., St. Louis, MO, USA) for 16 h served as a positive control, whereas cells cultured in complete medium for 24 h as a negative control. Cells were washed twice with cold PBS (Sigma-Aldrich Corp., St. Louis, MO, USA) and fixed with ice-cold 70% ethanol at −20 °C for 20 min. Subsequently, cells were treated with RNase A DNase&Protease-free (10 mg/mL) (Canvax Biotech, Spain) and incubated at 37 °C for 1 h before staining with PI solution (10 μg/mL) (Sigma-Aldrich Corp., St. Louis, MO, USA. After 30-min incubation at 4 °C percentage of cell cycle distribution in each phase was assessed by FC using the Beckman Coulter CytoFLEX.

### 4.6. Statistical Analysis

Statistical analysis was performed using the Mann–Whitney test in Sigma Plot (Systat Software, Inc.). Each of the analyzes in individual experiments was based on the results of three independent tests. The differences were statistically significant on the graphs as follows: * *p* < 0.05, ** *p* < 0.01, *** *p* < 0.001 versus negative control.

## 5. Conclusions

It may be concluded that universal bonding systems vary both in cytotoxic and genotoxic effect on human monocyte/macrophage peripheral blood SC cells. Prime&Bond Universal and Adhese Universal presented minimal toxic effect on human SC cells, while OptiBond Universal showed significant cytotoxic and genotoxic effect on SC cell line. Furthermore, only OptiBond Universal showed significant ability to induce apoptosis in SC cell line. None of the compounds showed an ability to arrest the cell cycle in G2/M phase. Studies concerning the cytotoxicity and genotoxicity of dental bonding systems vary in methodology and present different results. Therefore, there is a great need for establishing more reliable test protocols suitable for conducting standardized research on various dental materials including dental bonding systems. According to our results, studies concerning the biologic impact of dental bonding systems should be conducted using various tests including cytotoxicity, genotoxicity, apoptosis detection and cell cycle analysis studies. Colorimetric tests based on the cell metabolism may not provide a reliable result, since dental universal adhesives alter the color of the mixture which may have a significant impact on the obtained results. Thereby, the flow cytometry analysis in dental material research may provide more accurate evaluation and should be recommended by international standards such as ISO. Thus, the present study is the first that evaluate the dental bonding systems by multiple in vitro assays as well as suggest that currently used research techniques should be extended and incorporated into the standardization criteria.

## Figures and Tables

**Table 1:** Dental bonding systems.

Name	Manufacturer	Composition
OptiBond Universal	Kerr, Brea, CA, USA	Acetone (30–60%), HEMA (5–10%), glycerol dimethacrylate (1–5%), ethanol (5–10%)
Prime&Bond Universal	Dentsply Sirona, Charlotte, NC, USA	Phosphoric acid modified acrylate resin, multifunctional acrylate, bifunctional acrylate, acidic acrylate, isopropanol, water, initiator
Adhese Universal	Ivoclar Vivadent, Schaan, Liechtenstein	MDP, 3–10%, MCAP methacrylated carboxylic acid polymer, HEMA (10–25%), Bis-GMA (10–25%), D3MA (3–10%), 2-dimethylaminoethyl methacrylate (1–2.5%), camphorquinone (1–2.5%), ethanol (10–25%)

## Abbreviations

BPDM	biphenyl dimethacrylate
PENTA	dipentaerythritol pentaacrylate phosphoric acid ester
D3MA	decanediol dimethacrylate
HEMA	2-hydroxyethyl methacrylate
bis-GMA	bisphenol A-glycidyl methacrylate
MDP	methacryloyloxi-decyl-dihydrogen-phosphate
Phenyl-P	*N*-Phenyl-p-phenylenediamine
4-MET	4-methacryloxyethyl trimellitic acid
UDMA	urethane dimethacrylate
TEGDMA	triethylene glycol dimethacrylate
XTT	Cell Proliferation Kit II
MTT	Cell Proliferation Kit I
FC	flow cytometry
PI	propidium iodide
DAPI	4′,6-Diamidino-2-Phenylindole
SC	monocyte/macrophage peripheral blood cell line
PBS	Phosphate-buffered saline
